# The role of Ataturk University in data and publication production for sigmoid volvulus and ileosigmoid knotting

**DOI:** 10.12669/pjms.346.16113

**Published:** 2018

**Authors:** Sabri Selcuk Atamanalp, Esra Disci, Refik Selim Atamanalp

**Affiliations:** 1 *Prof. Sabri Selcuk Atamanalp, MD, Department of General Surgery, Faculty of Medicine, Ataturk University, Erzurum, Turkey*; 2 *Assist. Prof. Esra Disci, MD, Department of General Surgery, Faculty of Medicine, Ataturk University, Erzurum, Turkey*; 3 *Refik Selim Atamanalp, MD, English Medicine Program Faculty of Medicine, Ataturk University, Erzurum, Turkey*

**Keywords:** Sigmoid Colon, Ileum, Volvulus, Knotting, Literature, Web of Science

## Abstract

**Objective:**

Sigmoid Volvulus (SV) and Ileosigmoid Knotting (ISK) are rare intestinal obstruction forms, but Turkey is endemic for these diseases. The aim of this study was to evaluate the role of our publications in the world literature.

**Methods:**

We electronically searched Web of Science database to evaluate the manuscripts published during June 1966 to July 2018.

**Results:**

Total 788 manuscripts have been published by 289 different organizations (2.7 items per organization) around the world. Among them, 71 (9.0%) are from 31 different institutions in Turkey, while 37 manuscripts (4.7% of total and 52.1% of Turkish items), including 27 on SV and 10 on ISK, have been published by our clinic. Of our publication, 19 items (51.4%) were original articles, 10 (27.0%) were published in Turkish Journal of Medical Sciences, 30 (81.1%) were published over a period spanning from 2009 to 2018, and in 32 of which (86.5%) the corresponding author was Atamanalp.

**Conclusion:**

Arising from our 1,008-case SV and 80-case ISK experiences over a 52-year period between June 1966 and July 2018, our clinic has an important role in the data production and publication for SV and ISK. It seems that our clinic may provide several more documents in the years to come.

## INTRODUCTION

Sigmoid volvulus (SV) is the wrapping of the sigmoid colon around itself, while ileosigmoid knotting (ISK) is the complex volvulus of the ileum and sigmoid colon. Both SV and ISK cause intestinal obstruction.^1^,^2^ Although SV and ISK are relatively rare diseases worldwide, Eastern Anatolia, where we practice, is an endemic area for these diseases.^3^, ^4^ Our clinic has 1,008-case SV and 80-case ISK experiences over a 52-year period between June 1966 and July 2018. According to the literature documented in Web of Science,^5^ our SV series is the largest single-center series in the world, while our ISK series is one of the largest series. Consequently, our clinic has produced many publications on these topics over the years. In this report, we evaluated the literature published in different journals, which are indexed in the Web of Science.^5^

## METHODS

In the present study, we electronically searched the Web of Science database to find publications related to SV and ISK that are indexed in the Science Citation Index (SCI), Science Citation Index Expanded (SCI-E) and Emerging Sources Citation Index (ESCI) lists.^5^ Subsequently, we utilized manuscripts according to their publication addresses, topics, types, journals, years and authors.

## RESULTS

From the first publication by Ranger and Legros^6^ in 1970, 788 manuscripts have been published on SV and ISK and uploaded to the Web of Science.^5^ These manuscripts have been produced by 289 different organizations (2.7 items per organization) around the world. Among them, 71 (9.0%) are from 31 different institutions in Turkey. From the first paper by Alver et al.^7^ in 1993, 37 manuscripts (4.7% of total and 52.1% of Turkish items), including 27 on SV and 10 on ISK, have been published by our clinic arising from our 1,008-case SV and 80-case ISK experiences over a 52-year period between June 1966 and July 2018. The detailed data of our published manuscripts are presented in Table-I. As demonstrated, by our publications, 19 items (51.4%) were original articles, 10 (27.0%) were published in the Turkish Journal of Medical Sciences, 30 (81.1%) were published over a period spanning from 2009 to 2018, and in 32 of which (86.5%) the corresponding author was Atamanalp. The last author was also the most frequently published author in the world (with 33 manuscripts, 4.2% of the total items and 46.5% of the Turkish items) of articles discussing studies concerning SV and ISK.

**Table-I:** The detailed data of our manuscripts published in Web of Science.

Data	Result
***Type***	
Original article	19 (51.4%)
Correspondence	8 (21.6%)
Letter to the editor	5 (13.5%)
Review article	2 (5.4%)
Short communication	2 (5.4%)
Case report	1 (2.7%)
***Journal***	
Turkish Journal of Medical Sciences	10 (27.0%)
Colorectal Disease	8 (21.6%)
Pakistan Journal of Medical Sciences	4 (10.8%)
Techniques in Coloproctology	3 (8.1%)
ANZ Journal of Surgery	2 (5.4%)
Diseases of the Colon and Rectum	2 (5.4%)
Annals of Saudi Medicine	1 (2.7%)
Intestinal Research	1 (2.7%)
Journal of Clinical and Analytical Medicine	1 (2.7%)
Pediatric Surgery International	1 (2.7%)
Surgery	1 (2.7%)
Surgery Today	1 (2.7%)
The Eurasian Journal of Medicine	1 (2.7%)
World Journal of Surgery	1 (2.7%)
***Period***	
2009-2018	30 (81.1%)
1999-2008	6 (16.2%)
1989-1998	1 (2.7%)
1966-1988	0 (0.0%)
***Corresponding author***	
Atamanalp SS	32 (86.5%)
Alver O	2 (5.4%)
Disci E	1 (2.7%)
Karadeniz E	1 (2.7%)
Raveenthiran V	1 (2.7%)

## DISCUSSION

Turkey, particularly our region, Eastern Anatolia, is endemic for SV and ISK.^1^-^4^ The plausible causes of this interesting dispersion include a diet high in fibre and vegetables and the high altitude, which cause a redundant sigmoid colon with a narrow mesentery, an anatomical feature that is known to be a predisposing factor for SV and ISK.^8^-^10^ As a result of our comprehensive experience with SV and ISK, our clinic has produced many publications on these topics over the years. In addition to discussion of some well-known subjects, including epidemiology, clinical presentations, diagnoses, treatments and prognoses, some interesting and unfamiliar points have been brought to the attention of the academic and medical communities by our lab, including topics such as the effects of ganglion cell concentrations and colon dimensions in the development of SV,^11^,^12^ roles of CT and MRI in the diagnosis of SV and ISK,^13^,^14^ problems of the endoscopic examination of SV,^15^,^16^ principles of the elective treatment of SV^17^ and, finally, new classification systems of SV and ISK,^18^,^19^ which consist of treatment algorithms and prognosis-estimating attributes.

## CONCLUSION

Our clinic has played an important role in data production and publication for SV and ISK. Although a decrease has been observed in our SV and ISK incidences during recent years, it seems that our SV series may remain the highest numbers of subject, while our ISK series may remain one of the highest numbers of subjects leading to several more publications in the years to come.

### Author’s Contribution

**SSA:** Designed the study, collected and analysed the data, reviewed the literature and wrote the text.

**ED:** Collected the data.

**RSA:** Controlled the text.
